# Tumor suppressive Ca^2+^ signaling is driven by IP_3_ receptor fitness

**DOI:** 10.15698/cst2017.11.109

**Published:** 2017-11-01

**Authors:** Geert Bultynck, Michelangelo Campanella

**Affiliations:** 1 KU Leuven, Lab. Molecular and Cellular Signaling, Dep. Cellular and Molecular Medicine and Leuven Kanker Instituut (LKI), Campus Gasthuisberg O/N-I bus 802, Herestraat 49, BE-3000 Leuven, Belgium.; 2 Department of Comparative Biomedical Sciences, Royal Veterinary College, NW1 0TU, London, United Kingdom.; 3 University College London Consortium for Mitochondrial Research, University College London, WC1 6BT, London, United Kingdom.

**Keywords:** cancer, oncogenesis, tumor suppression, IP3R3, calcium signaling, PTEN, FBXL2, BAP1

Intracellular Ca^2+^ signals critically control a plethora of cellular functions, of which many impact cellular death and/or survival, processes often dysregulated in cancer 1. In healthy cells, Ca^2+^ signaling is employed for normal cell physiology and survival functions 2. Yet, when a cell is exposed to toxic stimuli or suffering from enduring cell stress, like irreparable DNA damage, the Ca^2+^-signaling toolbox can be rapidly switched from a "pro-survival" modus into a "pro-death" modus, thereby initiating demise pathways 3. The highly dynamic nature of Ca^2+^ signaling allows cells to swiftly response to stress and damage, preventing the survival of damaged cells and malignant transformation that eventually results in tumor formation. Alterations in the expression, activity and regulation of Ca^2+^-transport systems both at the plasma membrane and at organelles like the endoplasmic reticulum (ER) and mitochondria have been implicated in oncogenesis and neoplasia 1,4. These changes result in aberrant Ca^2+^-signaling events that could favor resistance to cell death, migration or senescence escape 5.

Over the last decade, we learnt that tight contacts and functional connections involving Ca^2+^ exchanges between the ER, the main intracellular Ca^2+^-storage organelle, and the mitochondria are pivotal for cell-fate decisions 3,6,7,8. These contact sites contain chaperone-coupled Ca^2+^-flux systems: the IP_3_ receptors (IP_3_Rs) at the ER side and the voltage-dependent anion channels (VDACs) at the mitochondrial outer membrane side 9. These are controlled/exploited by several cellular factors and regulatory proteins, including oncogenes and tumor suppressors 10,11,12. Basal Ca^2+^ fluxes between ER and mitochondria sustain anabolic pathways for mitochondrial metabolism, ensuring proper cell cycle progression 13. Yet, continued elevated ER-mitochondrial Ca^2+^ transfers result in loss of mitochondrial membrane integrity and release of apoptogenic factors 14. Tuned ER-mitochondrial Ca^2+^ transfer is therefore key to cells’ response to pro-apoptotic stimuli: the failure of which results in cell death resistance, as often observed in cancer cells 15,16. In fact, the efficacy of chemotherapeutic agents and photodynamic therapy depends on the ability of these agents to elicit ER-mitochondrial Ca^2+^ exchanges 15. In the context of apoptosis, previous work proposed unique roles for the type 3 IP_3_R isoform (IP_3_R3) 17 and type 1 VDAC isoform (VDAC1) 18 even though other IP_3_R isoforms can contribute to the initiation of cell death programs 19,20.

In some cells, the role played by the IP_3_R3 in pro-apoptotic Ca^2+^ transfers from ER to mitochondria might relate to its ability to preferentially partner with the VDAC1 complex 18. Notably, several tumor suppressors and oncogenes located at the ER membranes dodge ER Ca^2+^ homeostasis and dynamics 10,21. In general, tumor suppressors increase ER-mitochondrial Ca^2+^ fluxes, whereas oncogenes suppress ER-mitochondrial Ca^2+ ^fluxes. The actions of both tumor suppressors and oncogenes at the ER can involve changes of the steady-state ER Ca^2+^-filling state through modulation of sarco/endoplasmic reticulum Ca^2+^ ATPases (SERCA) and/or ER Ca^2+^-leak channels. For instance, during stress, the tumor suppressor p53 accumulates at the ER and enhances the Ca^2+^-pump activity SERCA, causing ER Ca^2+ ^overload and the likelihood for pro-apoptotic ER-mitochondrial Ca^2+ ^fluxes 22,23,24. The anti-apoptotic protein Bcl-2 increases IP_3_R phosphorylation and its sensitivity to IP_3_, enhancing the passive Ca^2+^ leak from the ER, lowering ER Ca^2+^ levels and so pro-apoptotic ER-mitochondrial Ca^2+^ fluxes 25. Several tumor suppressors and oncogenes have been identified as direct regulators of the IP_3_R, whereby tumor suppressors (like BRCA1, PTEN, PML) and oncogenes (like Bcl-2, PKB/Akt) that respectively promote and suppress the activity of IP_3_R channels by impacting their gating and consequently their open probability 10,21. Besides this direct regulation of IP_3_R gating, it is clear that total IP_3_R-protein levels impact Ca^2+ ^flux from the ER to the mitochondria and in turn cellular sensitivity to death as well. Insights are now available on IP_3_R degradation by ER-assisted and 26S proteasomal turnover after cell stimulation and IP_3_R activation 26. In such conditions, IP_3_Rs become ubiquitinated due to recruitment of the erlin1/2 complex and RNF170, an E3 ubiquitin ligase 27,28,29.

Hitherto, not much was known about the molecular mechanisms impacting basal IP_3_R turn-over and controlling their steady-state in stressed cells; equally, whether dysregulation of these mechanisms was involved in oncogenesis and/or tumor progression. Nevertheless, it is clear that IP_3_R levels do impact apoptotic sensitivity 20,30,31,32, and hence cell death and survival proteins were found implicated in regulating IP_3_R levels 33,34.

Recent work from Kuchay *et al.* revealed an unexpected role for the tumor suppressor lipid/protein phosphatase PTEN, an allele frequently lost in cancer 35 and well-known negative regulator of PKB/Akt signaling, stabilizing IP_3_R channels by protecting them from proteasomal degradation 36 (**Fig. 1**). This novel function adds to its recently discovered presence at the mitochondria-associated membranes (MAMs), where it contributes to cell death sensitivity by suppressing IP_3_R3-mediated Ca^2+^ fluxes 37. Independently of its catalytic activity, PTEN competes with the F-box protein FBXL2 (the receptor subunit of one of 69 human SCF (SKP1, CUL1, F-box protein) 38,39 to bind to IP_3_R3 channels, in particular to a region in the ligand-binding domain. Normal cells that express PTEN will have a low level of the IP_3_R3/FBXL2-complex formation, preventing the ubiquitination of IP_3_R3 channels and subsequent targeting to the proteasome. Consistently with previous observations 27,40, activation of cells with agonists increase FBXL2/IP_3_R3-complex formation and subsequent IP_3_R3 post-transcriptional regulation via ubiquitination similarly to RNF170-mediated ubiquitination of IP_3_Rs 27. Interestingly, FBXL2 activity itself is Ca^2+^ dependent, but antagonized by calmodulin 41. Hence, activation of IP_3_Rs may not only make these channels more susceptible for degradation by increased interaction with FBXL2 but release of Ca^2+^ itself through IP_3_Rs may trigger local activation of FBXL2 associated with IP_3_R3 leading to IP_3_R3 degradation. Notably, FBXL2 binding to its substrates (like cyclin D3) was found to occur via canonical calmodulin-binding motif thereby preventing ubiquitination. It is nonetheless likely that FBXL2 binding to IP_3_Rs does not occur by targeting its calmodulin-binding motif even though a role for calmodulin in regulating IP_3_R3/FBXL2-complex formation may not be ruled out. In any case, access to the degron region in the ligand-binding core of IP_3_R3 for FBXL2 is facilitated by deletion of the suppressor domain and loss of PTEN is associated with increased FBXL2 binding to IP_3_R3 and degradation of IP_3_R3, contributing to the apoptotic resistance of cells 36. In cancer cells lacking PTEN, FBXL2 knockdown could therefore restore IP_3_R3 levels and apoptotic sensitivity.

**Figure 1:**
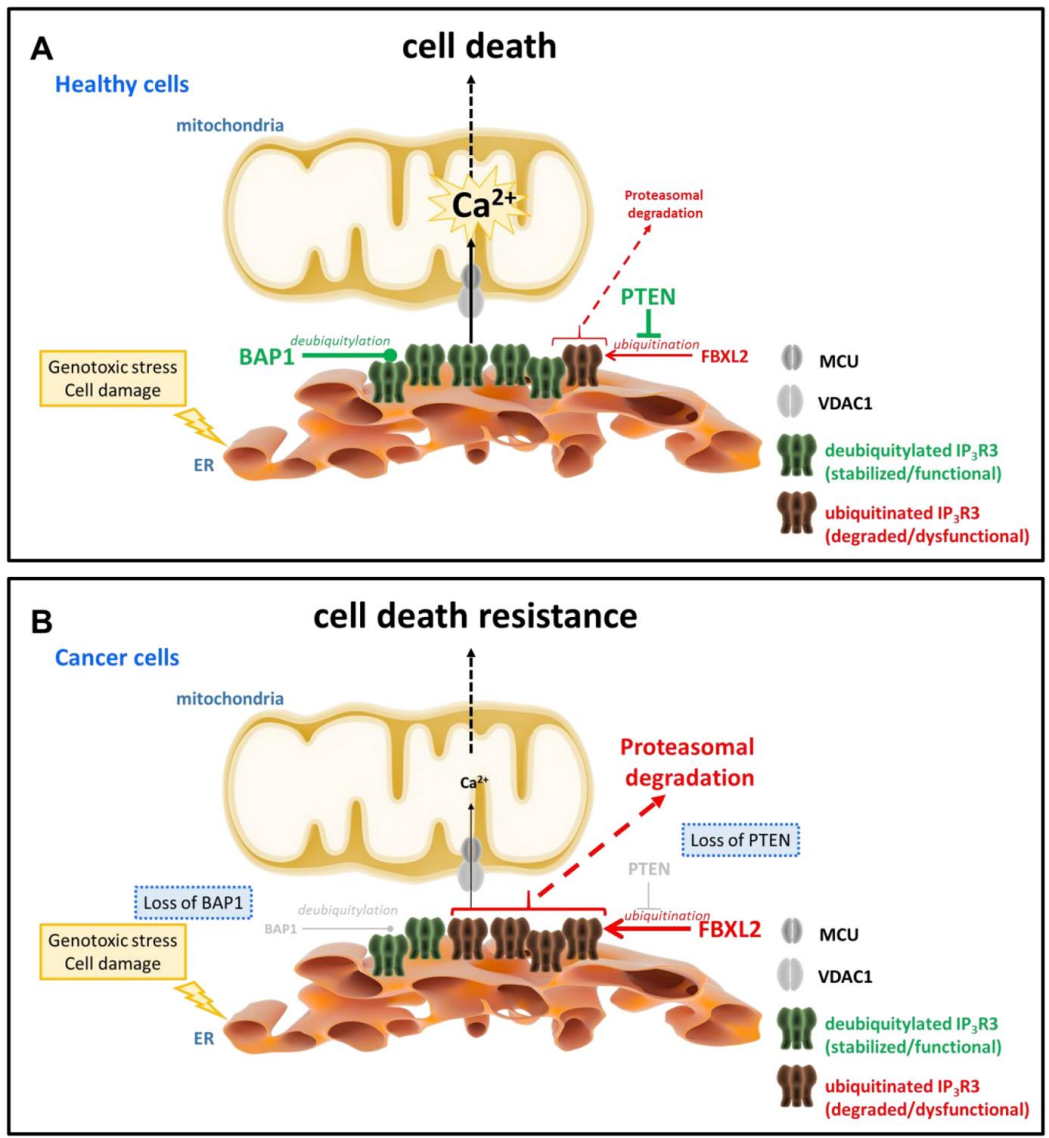
FIGURE 1: IP_3_R3 channels are targets for ubiquitination and deubiquitylation enzymes, whose activities are deregulated in cancer cells, thereby impacting IP_3_R3 stability and ultimately cell death sensitivity. **(A)** IP_3_Rs, located at the ER-mitochondrial interface, here particularly IP_3_R3, promote ER-mitochondrial Ca^2+^ fluxes, thereby enabling adequate cell death sensitivity in healthy cells undergoing genotoxic stress and cell damage, an important feature that prevents oncogenesis. The levels of IP_3_R3 channels are critical for this process and are dynamically regulated via ubiquitination. BAP1, a tumor suppressor with deubiquitylation enzyme activities and frequently mutated in cancers including mesothelioma, deubiquitylates IP_3_R3, preventing its proteasomal degradation and resulting in IP_3_R3 stabilization. In contrast, FBXL2, an F-Box protein with ubiquitin ligase activity, ubiquitinates IP_3_R3, directing it for proteasomal degradation and resulting in a decline in IP_3_R3 levels. Importantly, PTEN, another tumor suppressor that antagonizes Akt signaling and frequently mutated in a variety of cancers including prostate cancer, competes with FBXL2 for IP_3_R3 binding and thus antagonizes FBXL2-mediated ubiquitination of IP_3_R3, stabilizing IP_3_R3. In healthy cells, it is anticipated that the balance between deubiquitylation and ubiquitination of IP_3_R3 channels is favored towards deubiquitylated IP_3_R3, enabling sufficient IP_3_R3 channels to sustain adequate ER-mitochondrial Ca^2+^ fluxes in cells undergoing genotoxic stress and cell damage to engage cell death and eradicate pre-malignant cells with DNA damage. **(B) **Several cancer types display chromosomal aberrations with defects in the expression of several tumor suppressors, including a reduction in BAP1 levels and a loss in PTEN expression. As a consequence, both alterations will favor IP_3_R3 ubiquitination and degradation, since reduced BAP1 levels will lead to reduced deubiquitylation of IP_3_Rs and loss of PTEN will enable FBXL2 to mediate IP_3_R3 ubiquitination. As a consequence, the balance between ubiquitination and deubiquitylation of IP_3_R3s will shift towards ubiquitinated IP_3_R3, impairing ER-mitochondrial Ca^2+^ fluxes that are needed to engage cell death programs in response to genotoxic stress and cell damage. As such, cells will be able to withstand this cellular stress, resulting in their survival and proliferation despite accumulated DNA damage. This phenomenon is an early event in oncogenesis, enabling malignant cell formation and tumoral behavior.

Notably, authors show that by mutating the degron region in the ligand-binding domain in IP_3_R3 the FBXL2 binding and IP_3_R3 degradation were both prevented. Using a knockin approach in which wild-type IP_3_R3 was altered into a non-degradable IP_3_R3 mutant version (IP_3_R3^Q550A/F553A/R554A^), the authors could restore the rise of Ca^2+^ induced by photodynamic therapy and apoptosis in PTEN-negative cancer cells. This correlation was eloquently observed in human tumor samples beyond the convincing *in vivo* xenograft models, in which tumor cells expressing a non-degradable IP_3_R3 version or treated with geranylgeranyltransferase inhibitor (that prevents FBXL2 accumulation at ER membranes and activity, which depends on its geranylgeranylation 42) were greatly sensitized towards photodynamic therapy compared to tumor cells expressing wild-type (degradable) IP_3_R3 or untreated tumors.

It is important to note that FBXL2 has been previously implicated in cancer, but rather acting via the suppression of cell cycle progression and proliferation, as observed in lung tumors 43, leukemic cells 44, gastric cancer cells 45, and prevalently of tumor suppressive nature. Instead, in this work, the effects of FBXL2 on IP_3_R3 are tumor promoting by increasing IP_3_R3 degradation and making the cells more resistant to cell death 36. These effects are neutralized by PTEN, which prevents FBLX2 binding to IP_3_R3 channels. This interference by PTEN is likely selective for IP_3_R3 and not for other FBXL2 targets regulating the cell cycle. Nevertheless, the anti-cancer properties of FBXL2 activators like the small molecule BC-1258 46 will be adversely impacted by FBXL2-mediated IP_3_R3 degradation, likely limiting their application to PTEN-positive cancers. Vice versa*,* the previously discovered tumor suppressive properties of FBXL2 in cancer might be further boosted if FBXL2 could be selectively/subcellularly activated to pro-mote its cell-cycle targets while shielding IP_3_R3 for degradation.

In a separate study, Bononi *et al*. revealed a novel deubiquitylating enzyme that actually counteracts the level of IP_3_R3 ubiquitination, namely BRCA-associated protein 1 (BAP1) 47 (**Fig. 1**). BAP1 is a potent tumor suppressor, which protects against environmental stress and damage 48. Loss of one BAP1 allele either inherited or acquired during life has been associated with environmental stress-induced carcinogenesis, like UV light for uveal melanoma and asbestos for mesothelioma. Germline mutations in BAP1 resulting in aberrant/loss of BAP1 expression were associated with a high incidence of familial mesothelioma and uveal melanoma, while somatic mutations in BAP1 were found in sporadic mesotheliomas 49. Germline mutations in BAP1 greatly enhanced the sensitivity of mice to develop mesothelioma when exposed to asbestos 50. Recently, it has been shown in patient fibroblasts that loss of BAP1 displayed a metabolic rewiring towards aerobic glycolysis and reduced mitochondrial respiration associated with malignancy and carcinogenesis 51. BAP1 was therefore identified as a novel IP_3_R3-interacting protein that impacts its post-translational modification. BAP1 causes IP_3_R3 stabilization and prevents the channel to be degraded by the proteasome. Loss of only 1 allele in BAP1 is sufficient to protect cells from undergoing apoptotic cell death via suppressed Ca^2+^ release triggered by apoptotic stimuli like H_2_O_2_. An effect operated via the IP_3_R3 degradation due to a decreased BAP1-mediated deubiquitylation of IP_3_R3. As a consequence, exposure of cells being BAP1^+/-^ to DNA-damaging conditions will result in a higher percentage of cells surviving despite having damaged DNA. Such cells bearing genomic aberrations are at high risk for neoplastic behavior and oncogenesis, resulting in malignant cell growth and tumor formation. However, restoring IP_3_R3 in these cells could be an attractive strategy to reinstate cell death sensitivity of BAP1^+/-^ cells.

Based on these recent developments, it will be therefore important to examine the interplay between BAP1 and FBXL2. It is indeed not clear whether the increased IP_3_R3 ubiquitination observed when BAP1 levels are declined is mediated through FBXL2 and whether lack of BAP1 results in increased FBXL2 association with IP_3_R3 channels. In any case, by inhibiting FBXL2’s action on IP_3_R3 without inhibiting its action on cell cycle targets could be an attractive avenue to restore IP_3_R3 levels in these cancers. Another strategy can be boosting ER Ca^2+^-store loading in tumor cells by activating the SERCA pumps, thereby increasing the likelihood for ER-mitochondrial Ca^2+^ fluxes and restoring cellular sensitivity to apoptosis 22,52. Tumor-selective SERCA modulation is challenging but not impossible though, as evidenced by the ability to locally release thapsigargin in the vicinity of pancreatic tumor cells using peptide-coupled prodrugs that are enzymatically cleaved by prostate-specific factors 53. SERCA-activating approaches may be based on p53, recently proposed to activate SERCA activity in response to chemotherapy 22,23,24, although p53 is very frequently mutated in cancer. In wild-type p53 tumors, direct p53 activators might be of use. Other solace may come from SERCA-activating small molecules like CDN1163 54, provided these agents can be delivered to tumor cells while sparing healthy cells 52.

These recent papers highlight that deregulation of IP_3_R3 ubiquitination homeostasis not only impacts death and survival of cells but also contributes to the oncogenic behavior of cells with dysfunctional tumor suppressors by either (i) lacking PTEN 36 or by (ii) displaying deficiencies in BAP1 47. They underpin the emerging role of altered Ca^2+ ^signaling at the MAM level as a key event in apoptosis resistance that contributes early events associated with oncogenesis and tumor formation. Challenging will be the translation of these insights into anti-cancer therapies for which not only tumor-selective applications will be required but also further understanding in the selective targeting at the level of the IP_3_R3.

## References

[1] Monteith GR, Prevarskaya N, Roberts-Thomson SJ (2017). The calcium-cancer signalling nexus.. Nat Rev Cancer.

[2] Berridge MJ, Bootman MD, Roderick HL (2003). Calcium signalling: dynamics, homeostasis and remodelling.. Nat Rev Mol Cell Biol.

[3] Marchi S, Patergnani S, Missiroli S, Morciano G, Rimessi A, Wieckowski MR, Giorgi C, Pinton P (2017). Mitochondrial and endoplasmic reticulum calcium homeostasis and cell death.. Cell.

[4] Bultynck G, Parys JB (2017). Ca2+ signaling and cell death: Focus on Ca2+-transport systems and their implication in cell death and survival.. Cell.

[5] Ivanova H, Kerkhofs M, La Rovere RM, Bultynck G (2017). Endoplasmic Reticulum-Mitochondrial Ca2+ Fluxes Underlying Cancer Cell Survival.. Front Oncol.

[6] Marchi S, Patergnani S, Pinton P (2014). The endoplasmic reticulum-mitochondria connection: one touch, multiple functions.. Biochim Biophys Acta.

[7] Marchi S, Bittremieux M, Missiroli S, Morganti C, Patergnani S, Sbano L, Rimessi A, Kerkhofs M, Parys JB, Bultynck G, Giorgi C, Pinton P (2017). Endoplasmic Reticulum-Mitochondria Communication Through Ca2+ Signaling: The Importance of Mitochondria-Associated Membranes (MAMs).. Adv Exp Med Biol.

[8] La Rovere RM, Roest G, Bultynck G, Parys JB (2016). Intracellular Ca2+ signaling and Ca2+ microdomains in the control of cell survival, apoptosis and autophagy.. Cell Calcium.

[9] Giorgi C, Missiroli S, Patergnani S, Duszynski J, Wieckowski MR, Pinton P (2015). Mitochondria-associated membranes: composition, molecular mechanisms, and physiopathological implications.. Antioxid Redox Signal.

[10] Bittremieux M, Parys JB, Pinton P, Bultynck G (2016). ER functions of oncogenes and tumor suppressors: Modulators of intracellular Ca signaling.. Biochim Biophys Acta.

[11] Vervliet T, Clerix E, Seitaj B, Ivanova H, Monaco G, Bultynck G (2017). Modulation of Ca2+ signaling by anti-apoptotic Bcl-2 proteins at the ER-mitochondrial interface.. Frontiers in Oncology.

[12] Marchi S, Giorgi C, Oparka M, Duszynski J, Wieckowski MR, Pinton P (2014). Oncogenic and oncosuppressive signal transduction at mitochondria-associated endoplasmic reticulum membranes.. Mol Cell Oncol.

[13] Cardenas C, Foskett JK (2012). Mitochondrial Ca2+ signals in autophagy.. Cell Calcium.

[14] Giorgi C, Baldassari F, Bononi A, Bonora M, De Marchi E, Marchi S, Missiroli S, Patergnani S, Rimessi A, Suski JM, Wieckowski MR, Pinton P (2012). Mitochondrial Ca2+ and apoptosis.. Cell Calcium.

[15] Pedriali G, Rimessi A, Sbano L, Giorgi C, Wieckowski MR, Previati M, Pinton P (2017). Regulation of Endoplasmic Reticulum-Mitochondria Ca2+ Transfer and Its Importance for Anti-Cancer Therapies.. Front Oncol.

[16] Kerkhofs M, Giorgi C, Marchi S, Seitaj B, Parys JB, Pinton P, Bultynck G, Bittremieux M (2017). Alterations in Ca2+ Signalling via ER-Mitochondria Contact Site Remodelling in Cancer.. Adv Exp Med Biol.

[17] Marchi S, Marinello M, Bononi A, Bonora M, Giorgi C, Rimessi A, Pinton P (2012). Selective modulation of subtype III IP3R by Akt regulates ER Ca2+ release and apoptosis.. Cell Death Dis.

[18] De Stefani D, Bononi A, Romagnoli A, Messina A, De Pinto V, Pinton P, Rizzuto R (2012). VDAC1 selectively transfers apoptotic Ca2+ signals to mitochondria.. Cell Death Differ.

[19] Ivanova H, Vervliet T, Missiaen L, Parys JB, De Smedt H, Bultynck G (2014). Inositol 1,4,5-trisphosphate receptor-isoform diversity in cell death and survival.. Biochim Biophys Acta.

[20] Jayaraman T, Marks AR (1997). T cells deficient in inositol 1,4,5-trisphosphate receptor are resistant to apoptosis.. Mol Cell Biol.

[21] Akl H, Bultynck G (2013). Altered Ca2+ signaling in cancer cells: proto-oncogenes and tumor suppressors targeting IP3 receptors.. Biochim Biophys Acta.

[22] Bittremieux M, Bultynck G (2015). p53 and Ca2+ signaling from the endoplasmic reticulum: partners in anti-cancer therapies.. Oncoscience.

[23] Giorgi C, Bonora M, Missiroli S, Poletti F, Ramirez FG, Morciano G, Morganti C, Pandolfi PP, Mammano F, Pinton P (2015). Intravital imaging reveals p53-dependent cancer cell death induced by phototherapy via calcium signaling.. Oncotarget.

[24] Giorgi C, Bonora M, Sorrentino G, Missiroli S, Poletti F, Suski JM, Galindo Ramirez F, Rizzuto R, Di Virgilio F, Zito E, Pandolfi PP, Wieckowski MR, Mammano F, Del Sal G, Pinton P (2015). p53 at the endoplasmic reticulum regulates apoptosis in a Ca2+-dependent manner.. Proc Natl Acad Sci U S A.

[25] Oakes SA, Scorrano L, Opferman JT, Bassik MC, Nishino M, Pozzan T, Korsmeyer SJ (2005). Proapoptotic BAX and BAK regulate the type 1 inositol trisphosphate receptor and calcium leak from the endoplasmic reticulum.. Proc Natl Acad Sci USA.

[26] Wright FA, Wojcikiewicz RJ (2016). Chapter 4 - Inositol 1,4,5-Trisphosphate Receptor Ubiquitination.. Prog Mol Biol Transl Sci.

[27] Lu JP, Wang Y, Sliter DA, Pearce MM, Wojcikiewicz RJ (2011). RNF170 protein, an endoplasmic reticulum membrane ubiquitin ligase, mediates inositol 1,4,5-trisphosphate receptor ubiquitination and degradation.. J Biol Chem.

[28] Pearce MM, Wormer DB, Wilkens S, Wojcikiewicz RJ (2009). An endoplasmic reticulum (ER) membrane complex composed of SPFH1 and SPFH2 mediates the ER-associated degradation of inositol 1,4,5-trisphosphate receptors.. J Biol Chem.

[29] Wright FA, Lu JP, Sliter DA, Dupre N, Rouleau GA, Wojcikiewicz RJ (2015). A Point Mutation in the Ubiquitin Ligase RNF170 That Causes Autosomal Dominant Sensory Ataxia Destabilizes the Protein and Impairs Inositol 1,4,5-Trisphosphate Receptor-mediated Ca2+ Signaling.. J Biol Chem.

[30] Madesh M, Hawkins BJ, Milovanova T, Bhanumathy CD, Joseph SK, Ramachandrarao SP, Sharma K, Kurosaki T, Fisher AB (2005). Selective role for superoxide in InsP3 receptor-mediated mitochondrial dysfunction and endothelial apoptosis.. J Cell Biol.

[31] Khan MT, Bhanumathy CD, Schug ZT, Joseph SK (2007). Role of inositol 1,4,5-trisphosphate receptors in apoptosis in DT40 lymphocytes.. J Biol Chem.

[32] Assefa Z, Bultynck G, Szlufcik K, Nadif Kasri N, Vermassen E, Goris J, Missiaen L, Callewaert G, Parys JB, De Smedt H (2004). Caspase-3-induced truncation of type 1 inositol trisphosphate receptor accelerates apoptotic cell death and induces inositol trisphosphate-independent calcium release during apoptosis.. J Biol Chem.

[33] Schulman JJ, Wright FA, Kaufmann T, Wojcikiewicz RJ (2013). The Bcl-2 protein family member Bok binds to the coupling domain of inositol 1,4,5-trisphosphate receptors and protects them from proteolytic cleavage.. J Biol Chem.

[34] Pierro C, Cook SJ, Foets TC, Bootman MD, Roderick HL (2014). Oncogenic K-Ras suppresses IP3-dependent Ca2+ release through remodelling of the isoform composition of IP3Rs and ER luminal Ca2+ levels in colorectal cancer cell lines.. J Cell Sci.

[35] Beroukhim R, Mermel CH, Porter D, Wei G, Raychaudhuri S, Donovan J, Barretina J, Boehm JS, Dobson J, Urashima M, Mc Henry KT, Pinchback RM, Ligon AH, Cho YJ, Haery L, Greulich H, Reich M, Winckler W, Lawrence MS, Weir BA, Tanaka KE, Chiang DY, Bass AJ, Loo A, Hoffman C, Prensner J, Liefeld T, Gao Q, Yecies D, Signoretti S (2010). The landscape of somatic copy-number alteration across human cancers.. Nature.

[36] Kuchay S, Giorgi C, Simoneschi D, Pagan J, Missiroli S, Saraf A, Florens L, Washburn MP, Collazo-Lorduy A, Castillo-Martin M, Cordon-Cardo C, Sebti SM, Pinton P, Pagano M (2017). PTEN counteracts FBXL2 to promote IP3R3- and Ca2+-mediated apoptosis limiting tumour growth.. Nature.

[37] Bononi A, Bonora M, Marchi S, Missiroli S, Poletti F, Giorgi C, Pandolfi PP, Pinton P (2013). Identification of PTEN at the ER and MAMs and its regulation of Ca2+ signaling and apoptosis in a protein phosphatase-dependent manner.. Cell Death Differ.

[38] Skaar JR, Pagan JK, Pagano M (2009). SnapShot: F box proteins I.. Cell.

[39] Ilyin GP, Rialland M, Glaise D, Guguen-Guillouzo C (1999). Identification of a novel Skp2-like mammalian protein containing F-box and leucine-rich repeats.. FEBS Lett.

[40] Zhu CC, Wojcikiewicz RJ (2000). Ligand binding directly stimulates ubiquitination of the inositol 1, 4,5-trisphosphate receptor.. Biochem J.

[41] Chen BB, Coon TA, Glasser JR, Mallampalli RK (2011). Calmodulin antagonizes a calcium-activated SCF ubiquitin E3 ligase subunit, FBXL2, to regulate surfactant homeostasis.. Mol Cell Biol.

[42] Wang C, Gale Jr M, Keller BC, Huang H, Brown MS, Goldstein JL, Ye J (2005). Identification of FBL2 as a geranylgeranylated cellular protein required for hepatitis C virus RNA replication.. Mol Cell.

[43] Chen BB, Glasser JR, Coon TA, Mallampalli RK (2012). F-box protein FBXL2 exerts human lung tumor suppressor-like activity by ubiquitin-mediated degradation of cyclin D3 resulting in cell cycle arrest.. Oncogene.

[44] Chen BB, Glasser JR, Coon TA, Zou C, Miller HL, Fenton M, McDyer JF, Boyiadzis M, Mallampalli RK (2012). F-box protein FBXL2 targets cyclin D2 for ubiquitination and degradation to inhibit leukemic cell proliferation.. Blood.

[45] Li LQ, Pan D, Chen H, Zhang L, Xie WJ (2016). F-box protein FBXL2 inhibits gastric cancer proliferation by ubiquitin-mediated degradation of forkhead box M1.. FEBS Lett.

[46] Chen BB, Glasser JR, Coon TA, Mallampalli RK (2013). Skp-cullin-F box E3 ligase component FBXL2 ubiquitinates Aurora B to inhibit tumorigenesis.. Cell Death Dis.

[47] Bononi A, Giorgi C, Patergnani S, Larson D, Verbruggen K, Tanji M, Pellegrini L, Signorato V, Olivetto F, Pastorino S, Nasu M, Napolitano A, Gaudino G, Morris P, Sakamoto G, Ferris LK, Danese A, Raimondi A, Tacchetti C, Kuchay S, Pass HI, Affar EB, Yang H, Pinton P, Carbone M (2017). BAP1 regulates IP3R3-mediated Ca2+ flux to mitochondria suppressing cell transformation.. Nature.

[48] Jensen DE, Proctor M, Marquis ST, Gardner HP, Ha SI, Chodosh LA, Ishov AM, Tommerup N, Vissing H, Sekido Y, Minna J, Borodovsky A, Schultz DC, Wilkinson KD, Maul GG, Barlev N, Berger SL, Prendergast GC, Rauscher 3rd FJ (1998). BAP1: a novel ubiquitin hydrolase which binds to the BRCA1 RING finger and enhances BRCA1-mediated cell growth suppression.. Oncogene.

[49] Testa JR, Cheung M, Pei J, Below JE, Tan Y, Sementino E, Cox NJ, Dogan AU, Pass HI, Trusa S, Hesdorffer M, Nasu M, Powers A, Rivera Z, Comertpay S, Tanji M, Gaudino G, Yang H, Carbone M (2011). Germline BAP1 mutations predispose to malignant mesothelioma.. Nat Genet.

[50] Xu J, Kadariya Y, Cheung M, Pei J, Talarchek J, Sementino E, Tan Y, Menges CW, Cai KQ, Litwin S, Peng H, Karar J, Rauscher FJ, Testa JR (2014). Germline mutation of Bap1 accelerates development of asbestos-induced malignant mesothelioma.. Cancer Res.

[51] Bononi A, Yang H, Giorgi C, Patergnani S, Pellegrini L, Su M, Xie G, Signorato V, Pastorino S, Morris P, Sakamoto G, Kuchay S, Gaudino G, Pass HI, Napolitano A, Pinton P, Jia W, Carbone M (2017). Germline BAP1 mutations induce a Warburg effect.. Cell Death Differ.

[52] Chemaly ER, Troncone L, Lebeche D (2017). SERCA control of cell death and survival.. Cell.

[53] Doan NT, Paulsen ES, Sehgal P, Moller JV, Nissen P, Denmeade SR, Isaacs JT, Dionne CA, Christensen SB (2015). Targeting thapsigargin towards tumors.. Steroids.

[54] Kang S, Dahl R, Hsieh W, Shin A, Zsebo KM, Buettner C, Hajjar RJ, Lebeche D (2016). Small Molecular Allosteric Activator of the Sarco/Endoplasmic Reticulum Ca2+-ATPase (SERCA) Attenuates Diabetes and Metabolic Disorders.. J Biol Chem.

